# Rapid phase adjustment of melatonin and core body temperature rhythms following a 6-h advance of the light/dark cycle in the horse

**DOI:** 10.1186/1740-3391-5-5

**Published:** 2007-08-24

**Authors:** Barbara A Murphy, Jeffrey A Elliott, Dawn R Sessions, Mandi M Vick, Erin L Kennedy, Barry P Fitzgerald

**Affiliations:** 1 Maxwell H. Gluck Equine Research Center, Department of Veterinary Science, University of Kentucky, Lexington, KY 40546-0099, USA; 2 Department of Psychiatry and Sam and Rose Stein Institute for Research on Aging, University of California, San Diego, CA, USA

## Abstract

**Background:**

Rapid displacement across multiple time zones results in a conflict between the new cycle of light and dark and the previously entrained program of the internal circadian clock, a phenomenon known as jet lag. In humans, jet lag is often characterized by malaise, appetite loss, fatigue, disturbed sleep and performance deficit, the consequences of which are of particular concern to athletes hoping to perform optimally at an international destination. As a species renowned for its capacity for athletic performance, the consequences of jet lag are also relevant for the horse. However, the duration and severity of jet lag related circadian disruption is presently unknown in this species. We investigated the rates of re-entrainment of serum melatonin and core body temperature (BT) rhythms following an abrupt 6-h phase advance of the LD cycle in the horse.

**Methods:**

Six healthy, 2 yr old mares entrained to a 12 h light/12 h dark (LD 12:12) natural photoperiod were housed in a light-proofed barn under a lighting schedule that mimicked the external LD cycle. Following baseline sampling on Day 0, an advance shift of the LD cycle was accomplished by ending the subsequent dark period 6 h early. Blood sampling for serum melatonin analysis and BT readings were taken at 3-h intervals for 24 h on alternate days for 11 days. Disturbances to the subsequent melatonin and BT 24-h rhythms were assessed using repeated measures ANOVA and analysis of Cosine curve fitting parameters.

**Results:**

We demonstrate that the equine melatonin rhythm re-entrains rapidly to a 6-h phase advance of an LD12:12 photocycle. The phase shift in melatonin was fully complete on the first day of the new schedule and rhythm phase and waveform were stable thereafter. In comparison, the advance in the BT rhythm was achieved by the third day, however BT rhythm waveform, especially its mesor, was altered for many days following the LD shift.

**Conclusion:**

Aside from the temperature rhythm disruption, rapid resynchronization of the melatonin rhythm suggests that the central circadian pacemaker of the horse may possess a particularly robust entrainment response. The consequences for athletic performance remain unknown.

## Background

The suprachiasmatic nucleus (SCN) of the hypothalamus is the location of the master mammalian pacemaker that controls circadian rhythms of diverse physiological and behavioral phenomena, including rest-activity cycles, hormone secretion and body temperature. The circadian system provides endogenous timekeeping mechanisms that allow organisms to optimize survival by adaptively anticipating periods of activity and entraining physiological function to the solar day [1]. Accordingly, light is the primary time cue serving to synchronize circadian rhythms to the 24-hour period of the earth's rotation. Circadian entrainment mechanisms also ensure that behavioral, endocrine and other 24-hour rhythms of the body are phased or timed adaptively with respect to the environment. The SCN receives photic information via the retino-hypothalamic tract and transmits timing signals to synchronize peripheral clocks located throughout the organism [2].

In humans, disruption of the circadian timing system occurs in response to rotational shift work and transmeridian travel. Rapid displacement across multiple time zones results in a mismatch between the previously entrained program of the internal circadian clock and the new cycle of light and dark (LD), a phenomenon known as jet lag [3,4]. The most common symptoms of human jet lag, including malaise, appetite loss, fatigue and disturbed sleep, last until the circadian clock system adjusts to the new environmental conditions, re-establishing preferred phase relations among different rhythms and between these rhythms and the external environment [5,6]. Circadian desynchronization is of particular concern to athletes hoping to perform optimally at an international destination. Significant decreases in reaction times, cardiorespiratory functions and muscle strength have been reported following transmeridian travel in humans [7-10] with prolonged disturbance associated with easterly travel [11]. The consequences of jet lag are equally relevant for the equine athlete with the high frequency of air travel for international equestrian competition. However, there are no previous studies examining re-entrainment of the equine circadian system following a phase shift of the LD cycle, and the duration and severity of jet lag related circadian disruption is presently unknown. The precise cellular, organismal, and behavioural mechanisms underlying jet-lag induced reductions in athletic performance, human or equine, remain to be elucidated. Nonetheless, a reasonable first step in understanding the phenomenon may be to characterize the severity and longevity of measurable perturbations in normal circadian organization as can be observed by studying and quantifying the parameters of two or more well characterized circadian rhythms in relation to each other and to the LD cycles employed.

Because it is not possible to directly monitor the functional timing of the endogenous circadian clock, marker rhythms that measure clock output are commonly used to assess circadian phase position. Two common, useful and physiologically important markers of circadian phase have been the circadian rhythms of blood melatonin (MT) and core body temperature (BT). The 24-h melatonin profile provides a robust marker of circadian phase in humans [12,13] and has been used to provide reliable estimates of circadian adaptation to phase shifts [14-16]. Similarly, core body temperature is a commonly assessed marker of circadian phase [17-19] and has been used to determine rates of re-entrainment in humans [16] and rodents [20]. Both MT and BT have previously been shown to exhibit robust rhythms in the horse [21,22]. In the later study [22], seasonal changes in the duration of the nightly melatonin peak, including the circadian timing of melatonin onset and offset, was observed under an acutely extended 24 h dark period. An investigation under constant light (LL) confirmed that the various parameters of the equine body temperature rhythm are similar to those of several species of rodents [23].

The primary aim of this study was to evaluate the rates of re-entrainment of the equine MT and BT rhythms following an abrupt 6-h phase advance of the LD cycle, mimicking an eastward transmeridian journey across 6 time zones. We demonstrate that these two markers of circadian phase resynchronize significantly more rapidly than in humans and rodents in response to a similar phase shift, suggesting a more rapid and robust entrainment response of the central pacemaker in the horse.

## Methods

### Animals

Six healthy 2 year old mares (*Equus caballus*) of mixed light horse breed were used in this study. Animals were maintained outdoors under conditions of natural photoperiod prior to the experiment, which was conducted at the time of year (mid-September) corresponding to an approximate 12 h light/12 h dark (LD12:12) natural photoperiod (sunrise at 7:30 AM, sunset at 7.30 PM; longitude 84.5°W, latitude 38.1°N). Five days prior to the experiment, mares were housed in individual stalls in a light-proofed barn under a lighting schedule that mimicked the external photoperiod but with abrupt L/D and D/L transitions (LD 12:12). During the hours of light, stalls were lit by two 200 W light bulbs that produced an average light intensity of 350 lux at eye level. Access to water was *ad libitum *and feed was provided 4 times a day to prevent a conspicuous 24-h temporal cue [23]. The ambient internal barn temperature varied from 19–21°C during the experimental period. All procedures involving animals were approved by the Institutional Animal Care and Use Committee (IACUC).

### Experimental protocol

The day before initiation of sample collection, mares were fitted with indwelling jugular catheters. Beginning at 7:30 AM (ZT 0) on Day 0, and ending ~24 h later (ZT 0) blood samples were collected and rectal BT readings were taken by digital thermometer at 3-h intervals (9 samples). Blood samples were allowed to clot and kept overnight at 4°C. The next day, samples were centrifuged and the serum harvested and stored at -20°C until assayed for MT. Throughout the experiment, sampling during the hours of darkness was conducted with the aid of a dim red light from handheld flashlights. Mares were well accustomed to the sampling procedure and were only minimally disturbed by the presence of the sample collector. At each time point, samples were collected from mares in the same order with approximately 3 min intervals between animals. After experiencing one additional 12 h photoperiod at the usual time, an advance shift of the LD cycle was accomplished by ending the dark period 6 h early, resulting in lights on from 1:30 AM to 1:30 PM (ZT 18 became new ZT 0) starting on experiment Day 1 and continuing through Day 11. Beginning at lights on (1:30 AM/new ZT 0), blood sampling and BT readings were again taken at 3-h intervals for 24 h (ZT 0 to ZT 0) on alternate days for 11 days. The mares were consistently sampled in the same order at each sampling time with approximately 3 min intervals between animals. Sampling at ZT 0 was conducted immediately following lights-on and at ZT 12, immediately following lights-off.

### Melatonin radioimmunoassay (RIA)

Melatonin was measured by a commercial RIA kit (Alpco, Windham, NH) as described previously [24]. Briefly, a 1 ml serum aliquot was extracted according to the directions of the manufacturer and reconstituted in a buffer solution provided. Aliquots of the extracted samples were assayed in duplicate. Intra-assay coefficients of variation for low and high MT concentration pools were 14% and 5% respectively. The limits of detection of the assay averaged .5 pg/ml.

### Data Analysis

MT and BT time series data were analysed using a computer program based on the least squares cosine fit method of Nelson et al. [25]. For each mare and each experimental day (9 points, 24 h), this cosinor method gave estimates of 3 rhythm parameters: Acrophase (time of peak value of the fitted cosine function), Amplitude (half the difference between the minimum and maximum of the fitted cosine function) and Mesor (middle value of the fitted cosine curve representing the rhythm adjusted mean). A potential advantage of the cosinor method is that acrophase is calculated from the entire 24-h time series and represents the mean vector of the circular (24 h) distribution. Also, we chose this method because with 3 h sampling we did not have the temporal resolution to track changes in MT or BT rhythm phase adjustments using such markers as the onset (evening rise) of MT or the 24 h nadir in BT. One-way repeated measures ANOVA was used to assess significant changes in mean MT and BT values and changes in cosinor parameters over time on each individual day (pre- and post-shift). Two-way repeated measures ANOVA (Day × Time) was used to assess differences in MT and BT rhythms between Day 0 and Days 1–11 at the corresponding ZT times post-shift. Bonferroni post hoc tests were used to evaluate differences between time points where appropriate. Data was analyzed using Graph Pad Prism Version 4.0 for Windows (Graph Pad Software, San Diego, CA,) and are presented as the means ± SEM. A value of p < .05 was considered significant.

## Results

### Baseline body temperature (BT) and melatonin rhythm assessment

**Figure 1:**
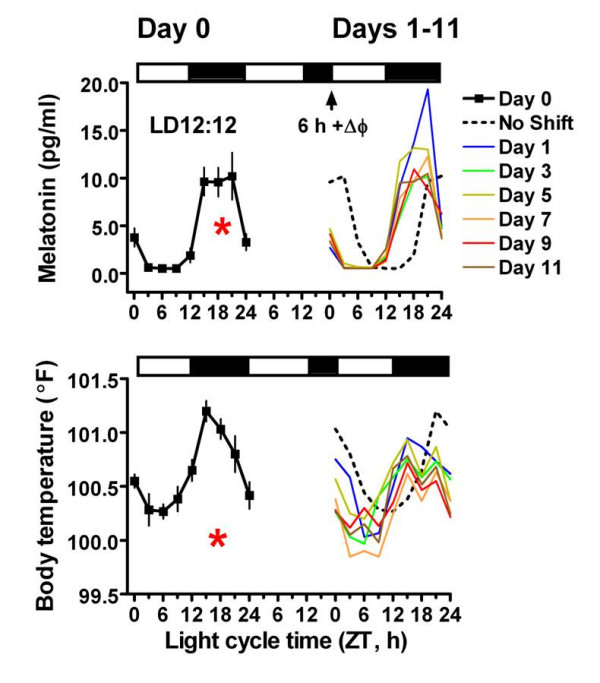
**Response of equine circadian melatonin (MT) and body temperature (BT) rhythms to a 6-h phase advance of LD12:12**. The LD cycle with 6-h phase advance is depicted above each graph with white bars representing light and black bars times of darkness. The abscissa represents light cycle time (ZT) in hours, where ZT 0 corresponds to lights on and ZT 12 to lights off of a 12 h photoperiod. Through Day 0 (curves at left) and for one additional day, lights were on 7:30 AM to 7:30 PM (ZT0-12). As indicated by the arrow, the photoperiod was advanced 6 h on Day1 (+Δφ = 6 h, from ZT18 to ZT 0) to give new lights on from 1:30 AM to 1:30 PM (Days 1–11). Baseline curves (mean+/-SEM) for MT (top) and BT (bottom) are plotted on the left from ZT 0 to ZT 24. Curves for Day 1 through 11 are plotted on the right with point symbols and SEM removed for clarity. The dotted lines retrace the Day 0 curve assuming no phase shift. Asterisks represent Day 0 mean acrophase times.

Individual cosine fits for both melatonin and BT profiles were significant for all mares on baseline Day 0 (Figure 1). The acrophase of the MT rhythm occurred at 1:35 AM (ZT 17.5, Table 1), almost exactly midway through the dark period, while the baseline BT acrophase occurred at 12:28 AM, (ZT 16.5) one hour and twenty minutes prior to the MT acrophase (Table 1). The latter is equivalent to a cosine nadir (bathyphase) at ZT 4.5 (12:28 PM). One-way repeated measures ANOVA (n = 6) revealed a significant variation in mean MT and BT values over time (p < .0001, respectively). MT values were low at ZT 0 and dropped to undetectable levels (< .5 pg/mL) at ZT 3, ZT 6 and ZT 9. Following a consistent but slight non significant rise at ZT 12, post hoc tests revealed that MT values at ZT 15, ZT 18 and ZT 21 were elevated significantly (p < .0001, respectively) with respect to daytime values. Inferred from ANOVA, the nadir in the BT rhythm was between ZT 3 and ZT 6 with significantly elevated temperatures occurring at ZT 15, ZT 18 and ZT 21 (p < .001, p < .001 and p < .01; respectively) as determined by post hoc comparison.

**Table 1:** MT and BT mean mesor, amplitude and acrophase values

**Melatonin**				**Temperature**		
**Day**	**Mesor (pg/mL)**	**Amplitude (pg)**	**Acrophase (CT)**	**Mesor (°F)**	**Amplitude (°F)**	**Acrophase (CT)**
0	4.61	5.66	1:35	100.63	0.45	12:01
1	5.7	8.45	8:36	100.55	0.45	8:33
3	4.16	5.13	8:50	100.45	0.4	6:07
5	5.63	7.38	8:08	100.57	0.38	6:00
7	4.35	6.0	8:31	100.23	0.45	7:58
9	4.23	5.65	8:45	100.37	0.28	6:34
11	4.46	5.78	7:57	100.38	0.36	6:37

Values are from baseline Day 0 and Days 1–11 post 6-h phase advance of LD cycle.

### Re-entrainment of MT rhythm post phase advance

**Figure 2:**
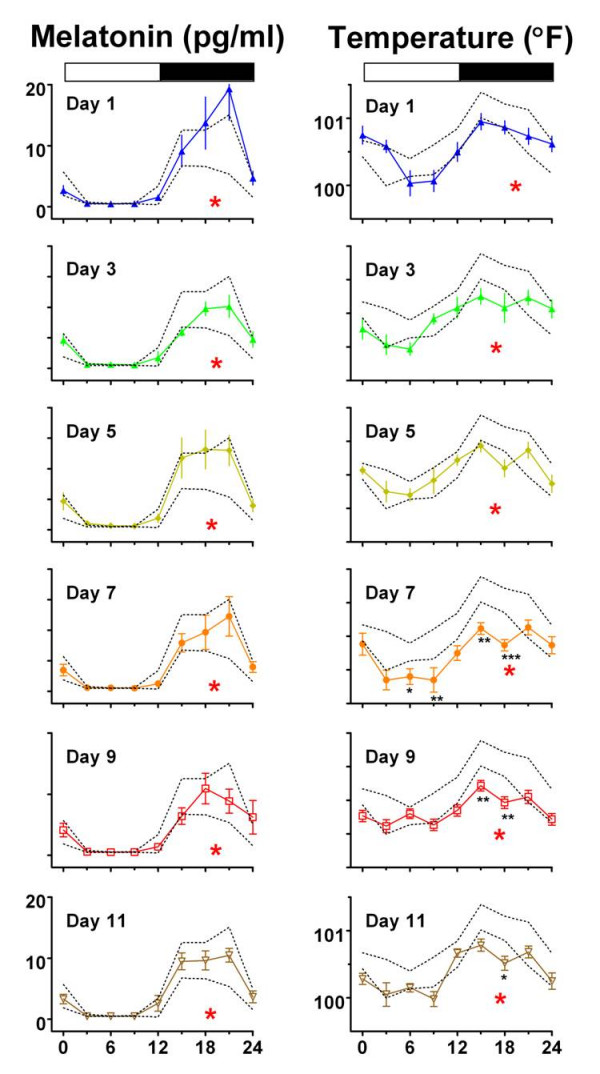
**Time course of reentrainment of MT (left) and BT (right) rhythms to a 6-h phase advance of LD12:12**. The Figure 1 curves for Days 1 through Day 11 are re-plotted in a vertical array to show mean+/-SEM in relation to the Day 0 curve, which has been advanced 6 h for comparison. Dotted lines connect the upper and lower 95% confidence limits of the phase shifted Day 0 means. Without exception, Day 1 through Day 11 melatonin curves closely paralleled the zeitgeber time adjusted (6 h advanced) baseline Day 0 curve. By contrast, BT curves on Days 7, 9 and 11, deviate significantly from baseline at individual ZT times marked by adjacent black asterisks (* p < .05; ** p < .01; *** p < .001). Large red asterisks beneath each curve represent cosine fitted acrophases (ZT), which for BT, are notably phase delayed in association with the waveform distortions on Days 7–11 (see Figure 3A). Other conventions as in Figure 1.

**Figure 3:**
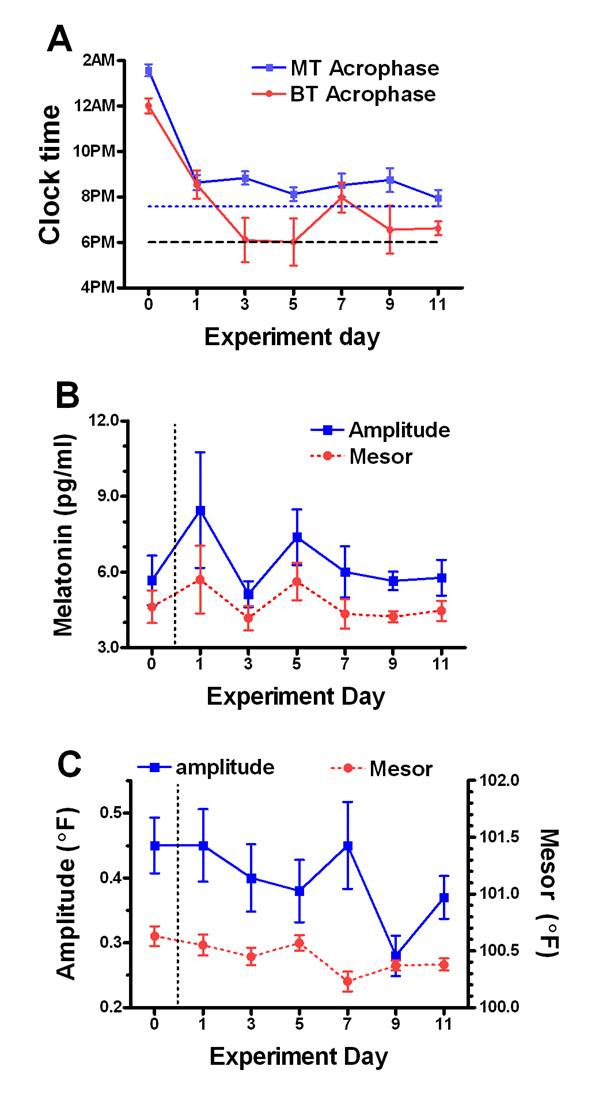
**Time course of changes in cosine acrophase, amplitude and mesor for equine MT and BT rhythms with a 6-h phase advance of LD12:12 between Day 0 and Day 1**. **A. **Change in acrophase clock time (mean +/- SEM) over Days 0 through 11 of the experiment. **B. **mean +/- SEM melatonin amplitude and mesor values. **C. **Means +/- SEM body temperature amplitude and mesor (right y axis) (Range in degrees centigrade: 37.8°C – 38.9°C). Dotted horizontal lines in A represent a 6 h phase advance relative to baseline (upper, MT; lower BT). Note that the consistent steep rise in MT between ZT 12 and 15 on all days suggests a rapid MT shift that is essentially complete (+ 6 h) on Day 1 and stable thereafter (Figure 2A).

Two-way repeated measures ANOVA of the MT data on Day 0 vs. Days 1–11 referenced to prevailing ZT times revealed no significant Day × Time interaction on any day post-shift (Figure 1 top panel and Figure 2A). This is consistent with a full 6 h advance of the rhythm to match the 6 h advance in the ZT scale. Thus, as on Day 0, there was a highly significant (p < .0001) effect of time of day on each day (1–11) with elevated levels occurring at the same ZT times as on pre-shift Day 0. Thus by ANOVA referenced to light cycle time (ZT), there was no significant alteration of the MT rhythm, and by inference the rhythm shifted in tune and in time with the LD cycle. The presence of this phase shift is further supported by ANOVA comparison of MT data on Day 0 vs Days 1–11 with the time series data referenced instead to unshifted sidereal (real world) clock time. This clock time based ANOVA revealed significant differences in mean MT values at 4.30 PM, 7.30 PM, 10.30 AM and 4.30 AM on Day 1 and at 4.30 PM, 7.30 PM, 1.30 AM and 4.30 AM on all other days post-shift. Figure 2A illustrates that circadian patterns of serum MT concentrations on each day post-shift were normal in waveform and fell within the 95% confidence limits of the phase shifted Day 0 means, with one exception. High MT levels were observed at ZT 21 on Day 1, when three of six mares exhibited abnormally high levels. While precise times of MT onset and offset could not be determined from the 3-h sampling protocol, a clear rise in MT levels was observed following lights-off (ZT 12) on all days post-shift. Similarly, significantly reduced MT values were detected directly following lights-on (ZT 24) on all days post-shift. There was a significant difference in the MT acrophase between Day 0 and Days 1–11 (p < .0001; Figure 3A). The acrophase shifted by five hours on Day 1 and remained stable thereafter. Repeated measures ANOVA revealed that neither the amplitude nor the mesor of the MT fitted cosine curves changed significantly on any of the post-shift days (Figure 3B).

### Re-entrainment of BT rhythm post phase advance

Following the 6 h phase advance of the LD cycle, there was no significant Day × Time interaction effect on the BT rhythm on Day 0 vs. Days 1, 3 and 5 as determined by two-way repeated measures ANOVA (Figure 1; bottom panel and Figure 2B). On these post-shift days, the shape of the BT rhythm was basically similar to that on pre-shift Day 0, but levels were generally lower and the curves not as sinusoidal as on Day 0. In contrast to the shift in the MT rhythm however, there was a significant Day × Time interaction (p < .05) with a Day effect manifest in comparing Day 0 vs. Days 7 and 9 (p < .001, p < .05; respectively). Also, while there was no significant interaction, there was also an effect of Day between Day 0 and Day 11. On these latter post-shift days, post hoc tests revealed significant differences comparing specific time points to the same ZT on Day 0, as denoted (asterisks) on Figure 2B. Post-shift disturbances in the waveform of the BT rhythm can also be visualized in the number of time points where the mean temperature falls below the 95 % confidence limits of the phase advanced Day 0 means (Figure 2B). Disturbances in waveform were also evident by the failure of individual cosine fits to reach significance in the latter days post-shift (data not shown). While not significant statistically, it is also worth noting the appearance of double peaks (ZT 15 and ZT 21) in the mean BT rhythm on all days following Day 1. There was a significant difference in the BT acrophase between Day 0 and Days 1–11 (p < .0001; Figure 3A). The acrophase shifted by three hours and twenty-eight minutes on Day 1 and completed the 6-h shift by Day 3. While the amplitude of the BT rhythm was statistically unaffected by the phase shift, there was a significant effect of experimental Day on the cosine mesor (p < .0001), with post hoc differences on Days 1, 5, 7, 9 and 11 (Figure 3C), reflecting the overall reduction in BT mesor (and mean) post-shift.

## Discussion

This is the first experiment of its kind to investigate the re-entrainment of two crucial circadian rhythm phase markers in the horse to an abrupt 6-h advance of the LD cycle. The null hypothesis was that following the 6-h phase shift of the LD cycle there would be no change in the phase of the MT and BT rhythms in relation to the LD cycle. Rejection of this null hypothesis would follow with evidence of acrophase times (ZT) on Days 1 through 11 that differ significantly from the corresponding Day 0 baseline acrophase. As no such differences could be demonstrated, there is, based on cosine analysis, no compelling evidence in the present study for phase-shift related transients in the entrainment of these two equine rhythms to a 6-h phase advance of an LD12:12 cycle. This surprising outcome is discussed further below in the context of human jet lag and in association with a more detailed consideration of the present data.

In humans, jet lag symptoms persist longer following an easterly rather than a westerly flight [26], theoretically, in part because the human clock displays an intrinsic free-running period greater than 24 h, making it more amenable to phase delays than to phase advances [11]. In addition, abrupt advances in the LD cycle have recently been shown to result in slower resynchronization of the molecular core clock components of endogenous oscillators within the rat SCN [3]. This finding suggests that a putative molecular mechanism may underlie the increased severity of physiological symptoms associated with eastward transmeridian air travel. An investigation of the equine circadian BT rhythm under constant light (LL) conditions demonstrated a circadian period of 24.2 h [23], suggesting that the equine circadian system, similar to that of humans, might have greater difficulty re-entraining to an advance shift of the LD cycle.

Our results indicate that in the horse, the MT rhythm, often regarded as the best marker of SCN pacemaker phase in humans [18], rapidly adjusts to a 6-h shift of the LD cycle. As has been previously observed in almost all mammalian species, including the horse [22], the duration of elevated MT levels on the baseline Day 0 reflected the duration of the scotophase. However, in contrast to human [27,28] and animal studies [29-31] that demonstrate gradual adaptation of the MT rhythm to an advanced photoperiod, the equine MT rhythm essentially appeared to complete the 6-h phase advance on the first post-shift day, as judged by the acrophase shift and by the 6-h advance in the evening rise of MT beginning at ZT 12. While we cannot exclude that more frequent sampling might have revealed transients not seen with 3-h sampling, the lack of distorted waveforms in the days post-shift strongly supports that stable re-entrainment had been achieved.

It is well known that MT production by the pineal gland increases significantly at night in both diurnal and nocturnal species and is a consequence of the high magnitude nocturnal rise in the enzyme serotonin-N-acetyltransferase [32]. The immediate rise in circulating levels of MT observed at ZT 12 on Day 0 is not surprising as a similar rapid rise has been observed in sheep immediately following exposure to darkness under conditions of normal 24-h entrainment [33]. However, the observation of a similar rapid rise at ZT 12 on Day 1 post-shift, a time corresponding with the midpoint of the subjective day in the pre-shift photoperiod, stands in stark contrast to what has been observed following phase advances in humans [15,28]. Van Cauter et al (1998) reported only a 2-h advance of the MT onset that occurred 6 h after lights-off when human subjects were exposed to the first afternoon dark period following a 6-h LD phase advance. Furthermore, the notable increase in MT from baseline levels at ZT 12 on all post-shift days highlights the rapid rise in equine melatonin production within minutes of the new dark period. Similar findings have been reported in the domestic pig [34]. Tast et al. report that an immediate rise in MT was observed in response to the first advanced scotophase when pigs were abruptly changed from a long day to a short day LD regimen. The significant reductions in MT levels at each sampling time directly following lights-on (ZT 0/ZT 24) concurs with the well-established ability of light to inhibit MT production in humans [35].

The rapid response of MT to the phase-advanced LD cycle can not distinguish true circadian entrainment from potential masking effects. For example, with the present data we can not know to what extent light suppression as opposed to a fully advanced circadian clock programming the MT decline, may have contributed to the precipitous drop in MT from ZT 21 to ZT 24 on all days. However, it is less likely that light masking (melatonin suppression) contributed to the abrupt rise in MT at ZT 12 – ZT 15 post shift. Importantly, in order to fully evaluate the physiological disruption caused by time-zone displacement in the natural environment, it is necessary to determine the effects that phase shifts exert on both the phase and waveform of circadian rhythms in the presence of the shifted LD cycle. Additional experiments will be necessary to reveal any contributions attributable to light masking, as opposed to true phase shifts of underlying circadian oscillators.

The unusually high melatonin levels that we observed at ZT 21 on Day 1 of the advanced LD cycle suggest an additive effect whereby the elevated MT production in response to the advanced scotophase overlapped with the previously entrained rise (perhaps not fully shifted) in MT associated with the pre-shift photoperiod. In the context of multiple SCN oscillators underlying the control of melatonin secretion, the increased secretion at ZT 21 could involve some oscillators advancing more than others to result in an increased overlap of oscillator outputs necessary to drive MT secretion occurring transiently at ZT 21. This is perhaps indicative of the combined controlling mechanisms of both the endogenous clock (SCN) and the prevailing LD cycle on MT secretion in response to a phase advance, as was similarly suggested for the pig [34].

In contrast to the rapid and apparently uncomplicated stable re-entrainment of the equine MT rhythm, re-entrainment of the BT rhythm was slower and not without disturbances in rhythm waveform. The acrophase of the BT rhythm was only partially advanced on Day 1 post-shift, requiring three days to completely adjust to the new LD cycle. However, while the acrophase of the fitted cosine curve completed the shift by Day 3, considerably faster than observations of BT resynchronization from similar studies in rodents [20,36], monkeys [37], and humans [16,38], the equine BT rhythm on subsequent post-shift days displayed significant waveform disturbances. The apparent decline in the robustness of the BT rhythm on post-shift days 7, 9 and 11 could suggest acute behavioural masking by the advanced LD cycle, but we know of no data to support this view. Alternatively, transient distortion of the BT rhythm post shift may reflect a phase misalignment of multiple SCN, or other, oscillators, which when in normal synchrony contribute to a more robust BT rhythm waveform. The double peaks at ZT 15 and ZT 21 which appeared on the latter days post-shift and in all individual rhythms (data not shown), though non-significant in ANOVA, could relate to such desynchrony, i.e. differential advances in BT related oscillators in the multi-oscillator SCN [39]

Aschoff first suggested that phase shifts of the LD cycle lead to decreases in amplitude [40]. The lower amplitude of the equine BT rhythm on the latter days post-shift may suggest a weakening of the association between the old zeitgeber and the circadian rhythm of BT. The finding of less stable, lower amplitude rhythms following phase shifts led to the postulation that this weakening helps hasten the shifting of the clock to the new zeitgeber.

The differences in re-entrainment rates between MT and BT rhythms in the horse likely reflect their independent regulation by different output pathways and different central circadian oscillators perhaps both within and outside the SCN [41]. Neural connections between the SCN and the paraventricular nucleus of the hypothalamus are thought to regulate melatonin secretion [42-44], whereas the dorsal subparaventricular zone has been identified as the SCN's neural target responsible for the circadian rhythm of BT [45]. Our results in the horse appear consistent with the idea that the output mechanism for MT may be more directly coupled to the central SCN pacemaker than the output mechanism for BT, in this species. This parallels a similar suggestion made with regard to the differential adaptability of BT and locomotor rhythms following an LD phase-advance in mice [36].

The recent discovery of two spatially distinct oscillators within the SCN [3] provides an alternative explanation for the observed differences in re-entrainment rates between MT and BT. Nagano et al. demonstrate differential resynchronization rates between the ventrolateral, photoreceptive region and the dorsomedial region of the SCN following abrupt LD shifts in rats. Clock gene expression in the ventrolateral region immediately responded to changes in environmental light. In this context, the regulatory mechanisms of the MT rhythm might be coupled to the light responsive oscillators within the SCN, thereby potentially explaining the more rapid resynchronization of this rhythm to the new LD cycle in the horse.

The difference between horses and other species in terms of adaptability to abrupt shifts in the LD cycle remains to be fully understood. It is interesting that an animal so dependent on circa-annual cues for seasonal breeding and survival of their young might be more rapidly responsive to circadian re-entrainment. However, we have previously demonstrated that clock gene expression in equine peripheral tissues is less tightly regulated than in other species [46]. As a large, visible, prey animal in a feral environment, with feeding and rest behaviors unrestricted to specific times in the 24-h natural LD cycle, it is conceivable that this observation may reflect a greater capacity for rapid re-entrainment to new environmental conditions.

Jet lag and shift work are considered one of the modern world's most prevalent challenges affecting human health and productivity in the work place [47-51]. Consequently, extensive resources continue to be employed in the design of potential treatment strategies that might facilitate or hasten re-entrainment and adaptation to the new LD conditions encountered following long flights or shifted work schedules. Our study reveals that in contrast to the human, the horse appears to possess a circadian pacemaker that is more amenable to rapid adjustment to a new photoperiod, suggesting in turn that their performance capacity at a new destination might be less compromised than in human athletes. Further investigation of this phenomenon could potentially provide new insights into re-entrainment mechanisms that might assist in the fight to conquer human jet lag.

## Competing interests

The author(s) declare that they have no competing interests.

## Authors' contributions

**BAM **conceived of the study, designed the study, collected and analysed the data and drafted the manuscript. **JAE **performed the cosinor analysis of data and assisted with figures, data analysis and drafting of the manuscript. **DRS **assisted in study design and data collection. **MMV **assisted in study design and data collection. **ELK **assisted in study design and data collection. **BPF **assisted in data collection and MT assays. All authors read and approved the final manuscript.
